# Can digital financial inclusion help reduce migrant workers’ overwork? Evidence from China

**DOI:** 10.3389/fpubh.2024.1357481

**Published:** 2024-06-05

**Authors:** Yuzheng Zhang, Yundong Li, Xugao Zhuang, Huan Liu, Yang Xu, Shuxian Zhang, Yueping Yan, Yalin Li

**Affiliations:** ^1^School of Ethnology and Sociology, Yunnan University, Kunming, China; ^2^School of Humanities and Management, Kunming Medical University, Kunming, China

**Keywords:** digital financial inclusion, overwork, migrant workers, individual differences, regional differences

## Abstract

**Introduction:**

Migrant workers in China are migrants from the rural to the urban areas who usually work in the cities and return to the countryside after a certain period. Due to China’s strict household registration system, they differ significantly from urban residents’ access to public services. However, at the same time, China’s workers are facing a severe phenomenon of overwork, and the group of migrant workers is even more hard-hit by overwork, which will cause various adverse effects on workers and society and should attract the attention of all sectors of society.

**Methods:**

This paper focuses on the impact of digital financial inclusion on the overwork of migrant workers. This study considered cross-sectional data containing 98,047 samples based on the 2017 China Migrants Dynamic Survey 2017 (CMDS) and China Municipal Statistical Yearbook after robustness tests and heterogeneity analysis using probit models.

**Results:**

(1) digital financial inclusion can effectively alleviate overwork among migrant workers; (2) the impact of digital finance on overwork is more significant for the new generation, digitized industries, and self-employed migrant workers; it is also more significant for the South, East, and small and medium-sized cities than for the North, the Midwest, and large cities; (3) job quality and income are crucial factors in how digital financial inclusion affects overwork among migrant workers. Digital financial inclusion can improve the quality of employment for migrant workers and alleviate overwork. However, the income substitution effect partially reduces the inhibitory impact of digital financial inclusion on overwork.

**Conclusion:**

Continuously promote the development of digital inclusive finance, improve laws and regulations, and protect the labor rights and interests of migrant workers. At the same time, vocational training and skills upgrading for rural migrant workers should be strengthened to improve the quality of their employment so that they can leave the secondary labor market and enter the primary labor market.

## 1 Introduction

The concept of overwork first originated in Japan with the enactment of the Labor Standards Law in 1947, which defines working more than 60 h per week as overwork and describes the condition of overwork as “a state in which fatigue due to activity cannot be recovered by rest including sleep” (1). According to data released by China’s National Bureau of Statistics (NBS), Chinese corporate workers’ average weekly working hours increased from 47.9 h in February to 48.7 h in March last year and 1.4 h from 47.3 h in March 2022. The data suggests that overworking in China is becoming a severe issue. There are significant differences in working hours between occupations (2). At the same time, some studies have shown that there may also be differences within the same field due to the work environment (3). In China, migrant workers typically work longer hours, averaging more than 55 h per week from 2010 to 2017, according to the National Bureau of Statistics of China. This figure is much higher than the 44 h stipulated in the “Labor Law of the People’s Republic of China” and far exceeds the working hours of workers in Europe and the United States (4). Migrant workers are at a higher risk of overworking compared to other groups. This is primarily due to their employment in the secondary labor market, characterized by high labor intensity and substitutability. The 2020 Migrant Workers Monitoring Survey Report reveals that over 70% of migrant workers are employed in construction, manufacturing, wholesale and retail trade, transport, accommodation, and food services, which are more labor-intensive and whose working hours are more flexible and closely linked to market changes, making it challenging to operate according to strictly defined hours. Migrant workers in these industries are at a higher risk of overworking. Existing studies indicated that formal employment sectors such as state organs and institutions, state-owned enterprises, collective enterprises, and foreign joint ventures have a higher proportion of moderate workers (5). These sectors typically belong to the primary labor market. The proportion of migrant workers in the formal employment sector is insufficient, leading to a “structural” overwork phenomenon. Additionally, the protection of migrant workers’ rights in China is relatively weak. As a mobile population, migrant workers often do not receive household registration of the place of inflow, and China’s existing urban–rural dual structure has resulted in significant disparities between urban and rural residents in healthcare, education, and social security (6). Migrant workers often lack social security or health insurance and may be forced to work longer hours or lose income when ill or job insecure. Therefore, they are more likely to be overworked. Third, the education level of migrant workers is low, and their legal awareness is weak. According to the China 2020 Migrant Workers Monitoring Survey Report, 86.8% of migrant workers have an education level of high school or below. Migrant workers are at a disadvantage in the labor market due to their lower levels of education, which results in lower job quality, and they are often in informal employment (4), which weakens their bargaining power over wages. They may have to increase their labor hours and income to maximize their benefits when employers prioritize economic gain. On the other hand, the low level of education weakens the legal awareness of migrant workers. Migrant workers often lack an understanding of their labor rights. They cannot protect themselves against employers violating their rights by not signing labor contracts or delaying wages. Additionally, weak legal constraints on working hours for informally employed workers in China exacerbate the issue of overwork (7).

Existing literature on the study of influencing the labor supply of migrant workers mainly includes the following aspects: first, economic factors. Existing studies believe that the primary purpose of migrant workers working outside the home is to obtain income that cannot be received in their hometowns, so for economic purposes, to increase wages as well as income, they will increase the level of labor supply (8, 9), the level of salary is an important reason that affects the labor supply of migrant workers, and the lower the wage rate of the migrant workers, the longer the working hours, Liu and Meng (10) found that the increase in the monthly income of the migrant workers will significantly increase their probability of falling into overwork, this phenomenon also exists in mobile individuals—second, social factors. Existing research suggests that some characteristics within the family affect the labor supply of migrant workers, such as the number of children (11); the more children the family has, the more adult males tend to increase their labor time, while adult females tend to decrease their labor time (12). This phenomenon also exists in other countries (13). Rent burden, migration patterns, and family living arrangements also have a particular impact on the labor supply of migrant workers. The heavier the rent burden of the family, the more serious the phenomenon of overtime labor of migrant workers, and compared to migrant workers who move with their families, those who have separated their father’s generation from the children’s generation have longer labor time and lower rate of return on their unit of time (14); compared to those who live in old neighborhoods, villages in the middle of the cities, and suburban areas, those who live in commercial housing and flat communities have lower labor time and lower rate of return on their unit of time. Migrant workers in commercial housing and balanced communities have higher wage rates than those in old neighborhoods and suburbs (15). In addition to the influence of the family, the health of the migrant workers will also have an impact on their labor supply. The higher the health of the migrant workers, the longer their labor time is, and the higher the probability of participating in the labor force (16). This influence has a degree of intergenerational effect; the infant’s health status also affects the labor supply of either the father or the mother (17, 18)—a third, the social system factor. Existing studies have found that although most migrant workers are concentrated in industries with “high-risk factors, high labor intensity, and high incidence of occupational diseases” (19), the government or enterprises can improve the ability of migrant workers to cope with risks using transferring payments and using social security, and avoiding migrant workers falling into a state of overwork by increasing their labor hours when they encounter troubles. Establishing a robust protection system, enabling migrant workers to join guilds, signing formal employment contracts, and purchasing insurance (20) can reduce the probability of migrant workers being overworked. These methods effectively protect migrant workers’ labor rights and interests, strengthen institutional support, and increase the stability of their work. It is important to note that these measures should be implemented objectively and without bias. Although previous studies have provided many explanations for the overwork of migrant workers, there are still some shortcomings. As mentioned above, the main reason for the overwork of migrant workers is that they are placed in the secondary labor market. If rural migrant workers cannot successfully enter the primary labor market, they will inevitably be subjected to overwork. The problem with previous research is that it did not discuss allowing migrant workers to escape the secondary labor market, so the proposed explanatory scheme may be invalid. For this reason, this study attempts to help migrant workers find ways to enter the primary labor market and eliminate overwork through the potential lens of digital financial inclusion. China has entered the digital economy era, and the entire society is undergoing digital transformation. Digital technology is promoting social change and rapid development of productivity. The digital economy will undoubtedly impact the employment environment of migrant workers and labor supply. It has been noted that the digital economy has the potential to create high-quality employment opportunities and further affect labor supply (21), which may help alleviate the overworked state of migrant workers. Digital financial inclusion, a crucial component of the digital economy, is also a significant means of achieving common prosperity in China and has had a certain impact on our society and economy. The essence of financial inclusion is to further expand the scope and accessibility of financial services through digital platforms and financial technology to realize the function of “inclusive services” that are more fair and equal (22). There have been studies on the impact of digital financial inclusion on migrant workers’ entrepreneurship (23), income growth (24), and multidimensional poverty (25), which provide a specific theoretical and empirical basis for the study. However, the impact of digital financial inclusion on the labor supply of migrant workers has rarely been discussed by scholars, mainly because of the lack of empirical verification analysis, so the specific role of digital financial inclusion on the overwork of migrant workers needs to be further explored.

In summary, the possible marginal contributions of this paper lie in the following: first, this paper incorporates digital financial inclusion into the analytical framework of migrant workers’ labor supply, empirically tests the relationship between digital financial inclusion and migrant workers’ overwork, and improves the relevant research on digital financial inclusion and migrant workers’ overwork, which is conducive to the further understanding of the problem of migrant workers’ labor supply. Secondly, through theoretical analysis and empirical tests, it tries to explore the specific mechanism of digital financial inclusion affecting the overwork of migrant workers and its heterogeneous characteristics to help the academic community enrich the corresponding theoretical foundation to assist in promoting the commonwealth of all the people in China’s high-quality development.

## 2 Theoretical analysis and research hypotheses

According to the theory of labor market segmentation, the labor market can be divided into primary and secondary (26). In the primary labor market, the labor force mainly relies on its knowledge, skills, and management for labor supply, and this market usually needs to be more competitive. In the secondary labor market, workers mainly rely on their physical strength for labor supply, and this market is generally competitive. Most migrant workers have low education levels and are not trained with professional skills to enter the primary labor market, so they mainly exist in the secondary labor market. In the secondary labor market, migrant workers lack bargaining power and increase their incomes, especially by extending their working hours, ultimately leading to overwork among migrant workers. In the past, it was difficult for traditional finance to help migrant workers because of the “Long Tail Effect.” Banks or financial institutions favored large multinational corporations or wealthy people to provide financial services and could not meet the financial needs of disadvantaged groups (27). Chris Anderson, editor-in-chief of Wired magazine, originally proposed the Long Tail Effect. By comparing the traditional and network entertainment industries, Anderson (28) found that due to cost and scale limitations, the traditional entertainment industry could only cover 20% of the mainstream population and ignored the tail behind it. However, network technology solves this problem, making it possible to meet the needs of more consumers under the premise of guaranteeing revenue. Recently, scholars have found a “long-tail effect” of digital financial inclusion on rural households (29). By providing financial services to migrant workers through digital means, digital financial inclusion can effectively reduce the “long-tail effect” and also alleviate the mobility constraints of migrant workers. The main reason why digital financial inclusion can help migrant workers mitigate overwork is because it breaks down labor market segmentation. Generally, migrant workers lack specialized knowledge and skills and are in a secondary labor market, where the trades and tools they work with are highly substitutable. With the help of digital financial inclusion, migrant workers can retrain their knowledge and skills, enhance their human capital, improve their “bargaining power,” cultivate professional knowledge and skills, and increase the probability of entrepreneurial behavior, which makes it easier for migrant workers to enter the primary labor market from the secondary labor market. Due to the non-competitive nature of the direct labor market, the wage rate of migrant workers increases, and they do not have to rely on increasing labor time to raise their income, thus objectively reducing the labor supply time of migrant workers and lowering the probability of overwork. In addition, the level of employment security and stability in the primary labor market is higher. The subjective socioeconomic status of migrant workers has improved, so they are endlessly unwilling to increase their labor time in exchange for compensation. They are more concerned about their careers and development, thus subjectively decreasing the labor supply time of migrant workers and lowering the probability of overwork. Based on the above analysis, this paper proposes research hypothesis 1.

*Hypothesis 1 (H1)*: Digital financial inclusion can have a disincentive effect by reducing the probability of overwork among migrant workers.

Employment quality refers to the degree of all the resources brought by workers’ work in the employment process in exchange for remuneration, mainly including income, employment environment, career prospects, social security, job satisfaction, and other aspects (30). Improving the employment quality is a response to the people’s growing aspirations for a better life and is also a specific requirement of China’s “14th Five-Year Plan.” Digital financial inclusion can fully release its universality and improve the employment quality of migrant workers. Specifically, digital financial inclusion can effectively enhance the financing constraints of small and medium-sized enterprises (31). Small and medium-sized enterprises are the main channel for the employment of migrant workers and provide more than 80% of jobs in China. However, under the dominance of the traditional financial system, which takes profit-making as the fundamental purpose, Small and medium-sized enterprises have been suffering from the shackles of financing difficulties for a long time; therefore, to cope with the constraints of financing, usually choose to reduce the cost and transfer the cost of human capital to the migrant workers, which makes migrant workers’ incomes remain low, employment security is not satisfied, and the employment quality is not satisfactory., unfulfilled employment security, and low employment quality. Compared with the traditional financial industry, digital financial inclusion expands the scope of financial services, releases the financing needs that cannot be met by the “long-tail effect” of traditional finance, helps to reduce the financing costs of small and medium-sized enterprises, promotes the optimal allocation of resources, enhances the vitality of the market, and promotes benign competition. This will help Small and medium-sized enterprises increase production capacity, increase revenue, improve regional technological innovation, and encourage economic growth (32). Economic growth will increase regional labor remuneration, attract more labor inflow, increase the labor costs of enterprises, and improve the employment environment. The migrant workers will also improve their job satisfaction after the increase in labor remuneration, which will promote the employment quality of the migrant workers. According to the efficiency wage theory, the higher the risk of unemployment, the more likely workers are to work hard to avoid job loss (33). On the one hand, improved employment quality can help migrant workers reduce the risk of unemployment, engage in jobs that require more professional knowledge and are less substitutable, enter the primary labor market, pay more attention to their career development, and get rid of the previous path of relying on increasing labor hours to raise labor compensation, thus reducing the supply of labor; on the other hand, improved employment quality can increase the remuneration of migrant workers, improve the subjective and objective socioeconomic status of migrant workers, and help migrant workers become more satisfied with the urban economy, and help migrant workers have a sense of identity in the city. After the identity of migrant workers in the city increases, they will actively imitate the behavior of citizens, and the labor time of citizens is relatively shorter. Hence, the labor time of migrant workers also declines, and the phenomenon of overwork has been alleviated. Based on the above analysis, this paper proposes the research hypothesis 2.

*Hypothesis 2 (H2)*: Digital financial inclusion can reduce the probability of overwork among migrant workers by improving their employment quality.

The theory of labor supply suggests that there is both an “income effect” and a “substitution effect” between income growth and labor supply (34, 35). In the front part of the labor supply curve, as income grows and the wage rate increases, the relative price of consuming leisure increases, so the “substitution effect” is greater than the “income effect, “and workers will reduce their consumption of leisure time while increasing the supply of labor time. At the back of the labor supply curve, after the wage reaches a certain level, workers will increase the consumption of leisure time and decrease the supply of labor time, and the “income effect” is greater than the “substitution effect.” However, for migrant workers, the overall income level is lower than that of urban residents. In the same labor market, they are more likely to be in the front part of the labor supply curve, so the “income effect” of migrant workers is greater than the “substitution effect” (36). Digital financial inclusion can effectively raise the income level of migrant workers and improve their labor supply. On the one hand, digital financial inclusion expands the scope of financial services, focuses on the “long-tail group, “helps to alleviate the financing constraints of migrant workers, provides financial support for migrant workers, promotes innovation and entrepreneurship of migrant workers, facilitates labor reproduction of migrant workers, reduces the incidence of poverty, and increases the income level of migrant workers (37). On the other hand, digital financial inclusion will improve the financial literacy of migrant workers, which is conducive to their financial investment. To meet the investment demand, migrant workers will improve their investment in human capital in the process of enhancing skills training and improving the human capital of migrant workers, which is conducive to the entry of migrant workers into the primary labor market and increases the level of income of migrant workers. As the “income effect” of migrant workers is greater than the “substitution effect, “the increase in migrant workers’ payments brought about by digital financial inclusion will increase the labor supply of migrant workers and reduce the inhibiting effect of digital financial inclusion on overwork. Based on the above analysis, this paper proposes research hypothesis 3.

*Hypothesis 3 (H3)*: The substitution effect of income weakens the disincentive effect of digital financial inclusion on the overwork of migrant workers.

## 3 Data and methods

### 3.1 Data sources

The data sources of this paper include three parts. The first part is the 2017 Digital Financial Inclusion Index of China’s prefectures and cities released by the Digital Finance Research Center research group of Peking University. The second part is the data from the annual large-scale national sample survey of the floating population, the China Migrants Dynamic Survey (CMDS), conducted by the National Health Commission since 2009. The content of the CMDS covers essential information on the floating population and its members, the scope and trend of mobility, employment, and social security, income and expenditure, residence, crucial public health services, marriage and family planning services, children’s mobility and education, and psychology and culture. The third part is the macro data at the city level, which is derived from the 2018 China Municipal Statistical Yearbook. In this paper, we use the prefecture-level city information of micro-individuals in the CMDS data to match the digital financial inclusion index and the *per capita* GDP and population density of prefecture-level cities. A series of control variables are selected, and after checking and eliminating the missing values, the number of individuals remaining is 98,047.

### 3.2 Methods

#### 3.2.1 Selection of model variables

The variable explained in this paper is the overwork of migrant workers. Overwork should include the consideration of both working hours and work intensity. Still, work intensity makes it difficult to conduct accurate quantitative research. Work intensity across occupations cannot be directly compared. Hence, the standard practice in the academic community is to use only the variable of working hours as a proxy for overwork; in summary, concerning the previous research (38), the weekly working hours of migrant workers more significant than 50 h are measured, and if the individual’s weekly working time is greater than 50 h, it is assigned a value of 1. If it is less than or equal to 50 h, it is given a value of 0.

The explanatory variable of this paper is digital financial inclusion, measured by the digital financial inclusion index released by the Digital Finance Research Center of Peking University, which is logarithmically processed because the original data is more significant than other values, which is prone to regression coefficient bias. The “Digital Financial Inclusion Index” consists of 33 indicators in three categories: breadth of coverage, depth of use, and degree of digitization. As of the third update, the index spans 2011–2020, covering 31 provinces, 337 cities above prefecture level, and about 2,800 counties in mainland China. The researcher collected the raw data of these 33 indicators and used the logarithmic efficacy function method for dimensionless processing, and then used the coefficient of variation method and the hierarchical analysis method for calculation (39).

This paper has two mechanism variables: the employment quality of migrant workers and their income. Employment quality refers to Fu Huang’s study (40). The payment of migrant workers, on the other hand, is measured by the logarithm of the individual monthly income of migrant workers and is supplemented by the logarithm of the monthly household income.

The control variables are mainly composed of four parts: demographic variables (gender, age, educational level, marital status), mobility characteristic variables (range, time), household characteristic variables (monthly income, Household size), and urban characteristic variables (GDP, population density). Descriptive statistics of the variables are shown in Table 1.

**Table 1:** Variable definition and descriptive statistics.

Variable	Definition and measure	Mean	Std. Dev.
Overwork	Weekly working hours >50 = 1, otherwise = 0	0.615	0.487
Digital financial inclusion (DFI)	Digital Financial Inclusion Index	5.536	0.097
Gender	Male = 0, Female = 1	0.428	0.495
Age	2017-the year of birth	35.742	9.739
Educational level	No schooling = 0, Primary school = 6, Junior high school = 9, High school = 12, Junior college = 15, undergraduate = 16, Graduate = 19	9.722	2.898
Marital status	Married = 1, unmarried = 0	0.838	0.368
Mobility range	Across province = 1, otherwise = 0	0.514	0.500
Mobility time	Length of flow into the local area	6.166	5.948
Monthly household income	The logarithm of monthly household income	8.682	0.556
Household size	Total number of family members	3.175	1.203
GDP (*per capita*)	The logarithm of GDP *per capita*	11.321	0.505
Population density	The logarithm of the number of persons per square kilometer	6.183	0.848

### 3.3 The models

According to the existing literature, and in conjunction with the research purpose of this paper, the Probit model is constructed as follows:


(1) $$Y_{ij}=\beta_{0}+\beta_{1}DFI_{j}+\beta_{2}X_{ij}+\mu_{j}+\varepsilon_{ij}$$

In Equation (1), Y represents the overwork of the migrant worker and is a binary variable; *i* represents different individuals, and *j* represents different cities. β0 is an intercept term, and β1 is the estimated coefficient of the effect of DFI on the overwork of the migrant worker. DFI is the explanatory variable of Digital Financial Inclusion, and *X* are other control variables, including individual and mobility characteristics. μ is the province fixed effect, and ε is the error terms.

## 4 Results

### 4.1 Baseline regression

Table 2 shows the effect of digital financial inclusion on the overwork of migrant workers, controlling for province-fixed effects in all regressions to eliminate the effects due to administrative divisions. Column (1) is the regression result of adding only digital financial inclusion on overwork, which shows that the coefficient of digital financial inclusion is −1.726, significant at the 1% level, which indicates that digital financial inclusion significantly reduces the probability of migrant workers falling into an overwork situation. Column (2) adds some demographic variables, mobility characteristics variables, and household variables, and the coefficient of digital financial inclusion is reduced but remains negatively significant at the 1% level. Column (3) adds urban characteristics variables, and the coefficient of digital financial inclusion remains significantly negative, indicating that digital financial inclusion can reduce the probability of migrant workers falling into overwork and inhibit the occurrence of overwork, initially verifying the research hypothesis H1 of this paper. From the regressions of the control variables concerning the demographic variables, the coefficient of the effect of gender on overwork is 0.165, which is significant at the 1% level, indicating that male migrant workers face a more severe overwork situation than female migrant workers. This finding is consistent with several current research (41). According to the traditional gender division of labor, male migrant workers are more engaged in the low-end manufacturing industry in the actual work, and they face tremendous survival pressure, so the situation of overwork is more serious. The influence of age on overwork shows a U-shape, with the probability of migrant workers falling into overwork increasing and then decreasing, which may be because in the period of youth, the increase in age of migrant workers brings about an increase in work experience and skills, and higher bargaining power in the labor market, and a shorter working time can bring a higher income; however, in the period of youth, the increase in age brings about an increase in work experience and skills, and a shorter working time can bring a higher income. However, as they gradually enter middle age, their physical condition deteriorates, their work efficiency decreases, and their bargaining power in the labor market decreases; to ensure that their incomes do not continue to decline, they can only gradually increase their work hours. Migrant workers with a high level of education have a lower probability of falling into overwork. This may be because migrant workers with a higher level of education have sufficient human capital and a more prosperous reserve of legal awareness, which can be used to improve labor supply. Those who are married have a higher probability of falling into overwork than those who are not. From the perspective of mobility characteristics, the longer the duration of mobility, the greater the probability of migrant workers falling into overwork. The overwork situation is more severe for cross-provincial migrant workers than those moving within the province. In terms of family characteristics, the larger the total family income and the size of the family, the more serious the phenomenon of overwork is. Regarding urban characteristics, the higher the *per capita* GDP, the less likely migrant workers are to fall into overwork, while population density’s effect is insignificant.

**Table 2:** Probit regression results.

Variables	Overwork		
	(1)	(2)	(3)
DFI	−1.726^***^ (0.062)	−1.337^***^ (0.064)	−0.836^***^ (0.141)
Gender		−0.165^***^ (0.008)	−0.178^***^ (0.009)
Age		−0.008^**^ (0.003)	−0.008^**^ (0.004)
Age squared		0.007 (0.004)	0.006 (0.005)
Educational level		−0.084^***^ (0.002)	−0.093^***^ (0.002)
Marital status		0.116^***^ (0.016)	0.112^***^ (0.016)
Mobility range		0.187^***^ (0.010)	0.170^***^ (0.010)
Mobility time		0.004^***^ (0.001)	0.004^***^ (0.001)
Household income (monthly)		0.068^***^ (0.008)	0.060^***^ (0.008)
Household size		0.064^***^ (0.004)	0.062^***^ (0.005)
GDP (*per capita*)			−0.114^***^ (0.021)
Population density			−0.015 (0.011)
Provincial fixed effects	YES	YES	YES
Pseudo-*R*^2^	0.0424	0.0811	0.0866
Observations	103969	103969	98047

**p < 0.05; ***p < 0.01; robust standard errors in parentheses.

### 4.2 Robustness test

This paper intends to conduct a robustness test to ensure the accuracy of the previous baseline regression results. The robustness test of this paper is mainly carried out through the following aspects: First, Changing the measurement of overwork; in previous studies, some scholars take the weekly labor time greater than 44 h (the labor time stipulated in the Labor Law of the People’s Republic of China) as a measure of overwork (4), or take the weekly labor time greater than 60 h as a criterion of severe overwork (42), so this paper will take these two criteria. Second, replace the model; the model used in this paper is the probit model; the logit model for regression will replace this part. The results are shown in Table 3.

**Table 3:** Robustness tests.

Variables	Overwork		
	Replace the explained variable	Replace the explained variable	Logit
	(1)	(2)	(3)
DFI	−0.650^***^ (0.011)	−0.246^***^ (0.018)	−1.402^***^ (0.235)
Province fixed	YES	YES	YES
Control variables	YES	YES	YES
Pseudo-*R*^2^	0.0755	0.0687	0.0873
Observations	98047	98047	98047

^***^p < 0.01; robust standard errors in parentheses.

According to Table 3, digital financial inclusion can significantly reduce the probability of overwork among migrant workers, whether by changing the measure of overwork or replacing the model, which suggests that the baseline regression results in this paper are robust.

### 4.3 Endogenous discussions

This paper discusses the relationship between digital financial inclusion and the overwork of migrant workers. The digital financial inclusion index, to a certain extent, is a macro variable; it is difficult to be affected by the overwork behavior of migrant workers as a micro individual, coupled with the fact that, according to the China 2022 Migrant Workers Monitoring Survey Report released by the National Bureau of Statistics, most of China’s migrant workers are concentrated in the low-end manufacturing. Labor supply in these industries has less impact on the financial sector, and it is difficult to affect the level of development of digital financial inclusion directly; digital financial inclusion and the overwork of migrant workers between the reverse causality are difficult to establish. But even so, considering that the possibility of endogeneity problems caused by omitted variables still exists, to ensure the rigor of study, this paper refers to the study of Yang (43) and selects the level of Internet development as an instrumental variable of digital financial inclusion, measured by the number of Internet access users in each prefecture-level city in 2017 (denoted as IV). In addition, this paper also adds the lagged one period of the digital financial inclusion index as the second instrumental variable (denoted as IV2), and the lagged one period of the digital financial inclusion index is directly related to the digital financial inclusion index without directly affecting the willingness to stay of the migrant workers. Hence, it satisfies the correlation and homogeneity conditions. The two instrumental variables are substituted into the Iv-probit model and tested for weak instrumental variables, and the results are shown in Table 4.

**Table 4:** Instrumental variables regression.

Variables	DFI	Overwork	DFI	Overwork
	(1)	(2)	(3)	(4)
IV	0.019^***^ (0.001)			
IV2			0.915^***^ (0.001)	
DFI		−2.052^***^ (0.479)		−1.170^***^ (0.151)
Province fixed	YES	YES	YES	YES
Control variables	YES	YES	YES	YES
AR chi^2^	18.40^***^		59.90^***^	
Wald chi^2^	18.39^***^		59.88^***^	
Observations	46425	32208	39612	17037

^***^p < 0.01; robust standard errors in parentheses.

According to Table 4, from the results of Column (1) and Column (3), the coefficients of the IV and IV2 are 0.019 and 0.915, which pass the significance test at 1% level. The values of AR and Wald are greater than 10, which indicate that the IV and IV2 have a significant positive effect on digital financial inclusion, and the results of Column (2) and Column (4) indicate that if the instrumental variables are introduced, the coefficients of digital financial inclusion will still significantly reduce the probability of migrant workers falling into overwork. By comparing the coefficients with those of the baseline regression model, it is concluded that the inhibitory effect of digital financial inclusion on the overwork of migrant workers may be underestimated if endogeneity is not taken into account.

### 4.4 Dimensional analysis

Digital financial inclusion is a comprehensive index with three dimensions: coverage, depth of usage, and degree of digitization. Therefore, in this section, we continue to study the role of these three dimensions on the overwork of migrant workers by substituting them into the probit model separately, and the regression results are shown in Table 5.

**Table 5:** Impact of three dimensions of digital financial inclusion on overwork.

Variables	Overwork		
	(1)	(2)	(3)
Coverage	−0.122^***^ (0.013)		
Depth of usage		−0.001^***^ (0.001)	
Degree of digitization			−0.002^***^ (0.001)
Province fixed	YES	YES	YES
Control variables	YES	YES	YES
Observations	98047	98047	98047

^***^p < 0.01; robust standard errors in parentheses.

According to the results in columns (1, 2), and (3) of Table 5, the impact coefficients of coverage, depth of usage, and degree of digitization of digital financial inclusion are −0.004, −0.001, and −0.002, respectively, which pass the test of significance at the 1% level. This indicates that all three dimensions of digital financial inclusion can effectively reduce the probability of migrant workers falling into overwork and inhibit the occurrence of overwork. However, regarding the specific impact size, the coverage role is greater than the depth of usage and degree of digitization. The possible reasons are: the coverage of digital financial inclusion can effectively alleviate the financing constraints of migrant workers, the effect of capital substitution of labor on migrant workers is stronger, the increase in the coverage of digital financial inclusion has well expanded the group of financial services, while the depth of usage means that financial services are provided to poorer groups. The first thing that poor groups satisfy is their own basic needs, and the capital substitution labor effect brought by the alleviation of financing constraints is even lower, so for migrant workers, the suppression of overwork by the breadth of coverage dimension of digital financial inclusion is stronger.

### 4.5 Heterogeneity test

#### 4.5.1 Individual heterogeneity

Individual heterogeneity is an important factor affecting labor participation or employment decisions, and previous studies have also proved that different groups and traits have different levels of labor supply (44). To comprehensively grasp the impact of digital financial inclusion on the overwork of migrant workers, this paper will conduct the individual heterogeneity test. After controlling for other variables, the regression results are shown in Table 6.

**Table 6:** Probit regression results for different individuals.

Variables	New generation	Old generation	Digital industry	Non-digital industry	Employed	Self-employed
	(1)	(2)	(3)	(4)	(5)	(6)
DFI	−0.999^***^ (0.184)	−0.515^**^ (0.217)	−2.234^***^ (0.521)	−0.737^***^ (0.146)	−1.568^***^ (0.203)	0.378^*^ (0.203)
Province fixed	YES	YES	YES	YES	YES	YES
Control variables	YES	YES	YES	YES	YES	YES
Observations	58230	39817	10410	87637	57399	40648

^***^p < 0.01, ^**^p < 0.05, ^*^p < 0.1; robust standard errors in parentheses.

Firstly, referring to Li et al. (45), according to the age of birth of migrant workers, they are divided into new-generation migrant workers and old-generation migrant workers, with 1980 as the benchmark. As can be seen from columns (1, 2) of Table 6, digital financial inclusion significantly inhibits the overwork of the new and old migrant workers. Still, the effect on the new migrant workers is much higher than on the old generation. The possible reasons for this are that compared to the older generation of migrant workers, the new generation of migrant workers use the Internet more frequently, are more digitally literate, are more receptive to new things, and are more likely to develop trust and engage in borrowing and lending behaviors when they come into contact with digital platforms, and that the new generation of migrant workers are more likely to start their own businesses and invest in their knowledge after obtaining a loan to improve their bargaining power in the labor market and enhance the quality of their employment; On the other hand, the older generation of migrant workers is limited by their age, their ability to accept new things is poor, their sensitivity to digital platforms is low, and their use of the Internet is not as skilled as that of the new generation of migrant workers, so they are unable to effectively utilize digital financial inclusion to increase their knowledge-based investment and entrepreneurship, and there exists a part of the phenomenon of the “digital divide, “which reduces the inhibiting effect of digital financial inclusion on the overwork, therefore, digital financial inclusion has a negative impact on the employment quality. Thus, the inhibiting effect of digital financial inclusion on the overwork of the new migrant workers is stronger than that of the old generation.

Secondly, referring to the related study of Zhang and Liu (46), this paper divides migrant workers into digital and non-digital industries according to the industry to which their main job belongs, and the regression results are shown in Table 6. Columns (3, 4) of Table 6 show that digital financial inclusion can effectively inhibit the overwork of migrant workers in both the digital and non-digital industries, but the impact on the digital industry is much larger than that on the non-digital industry. The possible reason is that for migrant workers in the digital industry, there are more “digital dividend.” They use computers, phones, and other digital terminals more frequently in their daily work, which requires a higher level of digital literacy, which forces migrant workers in the digital industry to continue to learn, strengthen their ability to accept new things, and enhance their ability to acquire and use information. So they can effectively use digital financial inclusion to expand reproduction and increase labor productivity, not rely on increasing labor time to obtain labor compensation and inhibit the emergence of overwork. In contrast, migrant workers in the non-digital industry can carry out their production and life without relying on digital terminals and devices, especially in the traditional manufacturing industry and the service industry, where the degree of digitization is relatively low, so digital financial inclusion is important to the migrant workers in the digital industry. Hence, digital financial inclusion has a stronger inhibiting effect on overwork for migrant workers in digital industries than in non-digital industries.

Finally, according to the employment status of migrant workers, this paper divides them into employed and self-employed groups (47), and the regression results are shown in Table 6. Columns (5, 6) of Table 6 show that the inhibitory effect of digital financial inclusion on migrant workers’ overwork is significant only among employed migrant workers, and for self-employed migrant workers, digital financial inclusion instead increases their labor supply and raises the probability of falling into an overworked state. The likely reason for this is whether or not to increase the labor supply is often the result of an autonomous choice by self-employed migrant workers instead of employed migrant workers. Self-employed migrant workers have certain knowledge and skills, higher levels of human capital, higher quality of employment, a stronger desire to increase their income, and a stronger “substitution effect” on income. In addition, self-employed migrant workers have easier access to traditional financial services in their production and business activities, so they do not need to rely on digital financial inclusion to obtain financing the role of digital financial inclusion is weaker for them, so the inhibiting effect of digital financial inclusion on the excessive labor of employed migrant workers is stronger.

#### 4.5.2 Regional heterogeneity

The results of regional heterogeneity are shown in Table 7, where columns (1) and (2) indicate that the inhibitory effect of digital financial inclusion on overwork is significant in the East and the Midwest. Still, specifically, the inhibitory effect of digital financial inclusion on overwork is greater in the Midwest migrant worker group. The possible reasons for this are that the labor market in the East is more perfect, the rights and benefits enjoyed by migrant workers are stronger than those in the Midwest, migrant workers in the East are not obliged to influence their labor supply through the channel of digital financial inclusion, and migrant workers in the Midwest receive more marginal benefits from it, which in turn has a stronger inhibitory effect on overwork. Columns (3) and (4) show that digital financial inclusion has a stronger disincentive effect on overwork for migrant workers in the North. The possible reasons for this are that relative to the South, the business environment in the North is poorer, the financing constraints of small and medium enterprises are stronger, the development of traditional finance is also weaker than that in the South, there is more unleashed demand for financing, and there is more room for the growth of digital financial inclusion. Therefore, digital financial inclusion has a stronger inhibitory effect on the overwork of migrant workers. Columns (5, 6) show that digital financial inclusion has a stronger inhibitory effect on the overwork of migrant workers in small cities. The possible reason is that small cities and medium enterprises face more serious financing constraints for their development. Therefore, small and medium enterprises shift the cost of human capital to migrant workers. The employment quality of migrant workers is lower than that of large cities. Digital financial inclusion has a stronger role in promoting the employment quality of migrant workers, which in turn has a stronger inhibiting effect on overwork.

**Table 7:** Probit regression results for different regions.

Variables	Overwork						
	Region					City size	
	East	Midwest	North	South	West	Large	Small
	(1)	(2)	(3)	(4)	(6)	(7)	(8)
DIF	−0.866^***^ (0.223)	−1.220^***^ (0.192)	−1.252^***^ (0.224)	−0.642^***^ (0.188)	0.194^***^ (0.038)	−0.424^***^ (0.203)	−1.393^***^ (0.372)
Province fixed	YES	YES	YES	YES	YES	YES	YES
Control variables	YES	YES	YES	YES	YES	YES	YES
Observations	50534	47513	38888	59159	38839	59208	50534

^***^p < 0.01; robust standard errors in parentheses.

### 4.6 Mechanism analysis

In the theoretical analysis part of the previous section of this paper, the hypothesis is put forward that digital financial inclusion may affect the overwork of migrant workers by affecting the two mediating variables of employment quality and income, so empirical tests are carried out in this section. The regression results are shown in Table 8. The results of columns (1) and (2) of Table 8 show that, controlling for all the control variables with the fixed effects of the province, the impact coefficient of digital financial inclusion on the overwork of migrant workers is 0.297 and significant at the 1% level. After substituting digital financial inclusion and employment quality variables into the model at the same time, the coefficients of digital financial inclusion and employment quality are −0.811 and −0.445, which are both significant at the 1% level, which indicates that the mechanism of digital financial inclusion to inhibit the generation of overwork of migrant workers by improving the quality of their employment indeed exists. The research hypothesis H2 of this paper is verified.

**Table 8:** Mediation effect results.

Variables	Employment quality	Overwork	Household income	Overwork	Income	Overwork
	(1)	(2)	(3)	(4)	(6)	(7)
DFI	0.297^***^ (0.033)	−0.811^***^ (0.144)	0.420^***^ (0.043)	−0.827^***^ (0.141)	0.350^***^ (0.080)	−0.820^***^ (0.140)
Employment quality		−0.445^***^ (0.007)				
Household income				0.048^***^ (0.010)		
Income						0.062^***^ (0.007)
Province fixed	YES	YES	YES	YES	YES	YES
Control variables	YES	YES	YES	YES	YES	YES
Observations	98047	98047	97796	97796	97796	97796

^***^p < 0.01; robust standard errors in parentheses.

The results in columns (3) and (4) show that the coefficient of the impact of digital financial inclusion on the total household income of rural migrant workers is 0.420 and is significant at the 1% level after controlling for all the control variables and province fixed effects. After adding the variable of total household income to the baseline regression, the coefficients of the impacts of digital financial inclusion and total household income are significant. Still, the coefficient of the impacts of total household income is positive. This indicates that digital financial inclusion will increase the household income of migrant workers. Still, the increase in household income has a certain “substitution effect” on migrant workers to obtain more income to increase the labor supply, weakening the inhibiting effect of digital financial inclusion on the overwork of migrant workers. Furthermore, this component is supplemented by including migrant workers’ income. The regression results are shown in columns (5) and (6). The results show that the digital financial inclusion on the personal income of farmers is significantly positive. After adding it to the benchmark regression, the impact coefficient of the personal income of migrant workers on overwork is also significantly positive. This indicates that like household income, personal income also produces a certain “substitution effect”; digital financial inclusion will increase the personal income of farmers and reduce the inhibitory effect of digital financial inclusion on the overwork of migrant workers. In conclusion, both the household income and personal income of migrant workers have a certain “substitution effect,” which weakens the inhibition effect of digital financial inclusion on the overwork of migrant workers. The research hypothesis H3 of this paper is verified.

## 5 Discussion

The research in this paper shows that the development of digital financial inclusion can reduce the probability of farmers falling into overwork. Moreover, empirical results show that digital financial inclusion can improve the employment quality of migrant workers. This result is similar to previous studies (48). More critically, evidence from Italy shows that credit constraints affect workers’ labor supply, and workers subject to credit constraints work more extended hours (49). The study from Italy, combined with this paper, provides complete evidence that financial market development has a role in the labor market. Groups with access to financial services are better off, and those that do not have access to financial services are worse off. According to the data of 2021, the number of migrant workers in China is 290 million, which is a massive group of people, and it is of great practical significance to pay attention to their working conditions. Regarding overwork, previous studies have focused too much on the consequences it leads to or the cultural, social, and economic factors that affect it and have not considered the impact caused by the financial market. In studies on financial markets, the social significance is seldom examined, and researchers always look at the benefits at the corporate level. The issues discussed in this study have been neglected to some extent. Of course, this study has some limitations. Instead of using tracking data for the study, only cross-sectional data were used, which may have impacted the results.

## 6 Conclusion

### 6.1 Conclusion of the research

Based on the 2017 China Migrants Dynamic Survey at the micro level (CMDS) and the matched data at the macro level of prefecture-level cities, this paper establishes a probit model to empirically study the impact of digital financial inclusion on the overwork of migrant workers. The following conclusions are drawn after in-depth research on the heterogeneity and impact mechanism. First, digital financial inclusion significantly reduces the probability of migrant workers falling into overwork, and this result remains robust after adding each control variable and controlling for area fixed effects. All three dimensions of digital financial inclusion produce an inhibitory effect on migrant workers’ overwork. Still, the inhibitory effect of the breadth of coverage is greater than that of the depth of use and the degree of digitization. Second, the effects of digital financial inclusion on the overwork of migrant workers are individually and regionally heterogeneous: the inhibitory effect of digital financial inclusion on the overwork of migrant workers is more robust in the central and western parts of China, the North, and the small cities than in the East, the South, and the large cities; and the inhibitory effect of digital financial inclusion on the overwork of the new generation, the digitized industry, and the self-employed migrant workers is more potent than that of the old generation, the non-digitized industry, and the employed migrant workers. Finally, employment quality and income are essential mechanisms for the impact of digital financial inclusion on the overwork of migrant workers. Digital financial inclusion can improve the employment quality of migrant workers and inhibit the generation of overwork, but the “substitution effect” of income will reduce part of the effect.

### 6.2 Policy recommendations

Based on the above conclusions, the following recommendations are put forward: (1) Continuously promote the development of digital financial inclusion, alleviate financing constraints, realize equality of opportunity between migrant workers and urban residents in the process of entrepreneurship and employment, enable migrant workers to have the ability and capital to make intellectual and financial investments, improve the financial literacy and human capital level of migrant workers, and encourage the cooperation between traditional finance and digital financial platforms, so that the “market sinking, “especially at the depth of usage as well as degree of digitization, to expand the scope of services. At the same time, it will increase the construction of digital infrastructure, prevent and solve the problem of the “digital divide” problem, and effectively utilize the “digital dividend” to benefit migrant workers (2) It is necessary to increase the protection of the legitimate labor rights and interests of rural migrant workers, to form adequate supervision of enterprises, to ensure that rural migrant workers enjoy the legal right to rest and leave and compensation for overtime work, and to compel enterprises to sign formal and legal labor contracts with rural migrant workers, to provide legal protection for rural migrant workers to safeguard their rights and interests. In addition, the government should increase legal aid for migrant workers to help migrant workers in disadvantaged positions safeguard their labor rights and interests (3) Strengthen the vocational training and skills upgrading of migrant workers to improve the employment quality so that they can leave the secondary labor market, where they can only rely on increasing their labor supply time in exchange for labor compensation, and enter the primary labor market. Considering the heterogeneity, training especially for the South, the East, the large cities, as well as for the older generation, non-digitized industries, and self-employed migrant workers, will help them better adapt to the demands of modern industries, improve employment opportunities, and alleviate the pressure of overwork. At the same time, a sound social security system will be established to improve protection capacity. The construction of a nationally unified social security platform and the use of digital means to promote the equalization of social security services will help migrant workers improve their ability to cope with risks, reduce the probability of their overwork occurring to achieve high-quality development and promote shared prosperity.

## Data availability statement

The data analyzed in this study is subject to the following licenses/restrictions: The China Migrants Dynamic Survey (CMDS) Data is a large-scale survey sponsored by the National Health and Wellness Commission of China, and use of the data requires access to and approval of this website. Requests to access these datasets should be directed to https://www.chinaldrk.org.cn/wjw/#/data/classify/population.

## Author contributions

YZ: Conceptualization, Methodology, Writing – review & editing, Writing – original draft. YuL: Conceptualization, Methodology, Writing – review & editing, Formal analysis. XZ: Conceptualization, Formal analysis, Software, Writing – review & editing. HL: Conceptualization, Methodology, Writing – review & editing. YX: Supervision, Conceptualization, Writing – review & editing. SZ: Data curation, Writing – review & editing. YY: Resources, Writing – review & editing. YaL: Funding acquisition, Supervision, Software, Writing – review & editing.
